# Kafka and Asking the Right Question at the Right Time

**DOI:** 10.1093/function/zqab013

**Published:** 2021-02-26

**Authors:** Ole H Petersen

**Affiliations:** School of Biosciences, Cardiff University, Wales, CF14 0ES, UK

I was recently invited to give a talk at a webinar organized by the World Academy of Art & Science and this prompted me to think again about a topic that has always fascinated me, namely, the similarity and difference in creativity required by these two branches of human intellectual endeavor or, as CP Snow named them, “the two cultures.”^1^ In both cultures, a central issue is how and when to ask the right question. One of the most intriguing dissections of this issue can be found in Chapter 9 of Franz Kafka’s incomplete novel “Der Prozess” (The Trial),^2^ unquestionably one of the most important and influential books of the 20th century. The novel has been analyzed by numerous literary scholars, theologians, and philosophers, who have interpreted it in many different ways and from many different perspectives.^3^

The central part of Chapter 9 is the parable “Before the Law,” told by a priest to the novel’s main figure, Joseph K. Briefly, a man from the country begs for entry to the Law. The doorkeeper tells him that he cannot be admitted at the moment, but says that it is possible that he could be allowed to enter later. In the following days, months, and years, the man repeats his question about whether he can now enter and is each time told that he cannot be admitted now. He is warned not to try to enter without permission, as the doorkeeper emphasizes that he is mighty, but that there are even mightier doorkeepers behind him. The man waits and ask and then waits again, and as the years go by, he becomes more and more fixated on the doorkeeper, noticing, for example, the fleas in his coat and even asking the fleas to help him get permission to enter. Having grown old and very weak, sensing that his end is near, he finally asks the doorkeeper why, in these many years, nobody else has come to seek entrance to the Law. The doorkeeper understands that the man is dying and answers “No one but you could gain admittance through this door, since this door was intended only for you. I am now going to shut it.”^2^ Joseph K’s immediate reaction to the story is that the man has been deceived by the doorkeeper, who should have given him the crucial information, namely, that the door was meant for him only, much earlier. However, the priest points out that the man only asked the doorkeeper about this at the very end of his life and then was given the correct answer.

There are of course many and deeply philosophical issues in this parable that has always eluded any definitive interpretation.^3^ However, from a humble and practical scientist’s point of view, one way of looking at this story may be to consider that the man from the country was so intimidated by the doorkeeper that he forgot to ask the crucial question and did not dare to enter without permission. He lost confidence in his ability to enter the Law and became so fixated on the doorkeeper, and unimportant details concerning his appearance, that he could not think straight. No doubt, many scientists have not asked the right question at the right time, that is, done a critical experiment, because they did not think it was feasible and because they became fixated on the hurdles, seeing only arguments against the likelihood of the experiment succeeding, rather than having faith in their ability to overcome the obstacles.

I have experienced such a situation myself. In April 1983, Yoshio Maruyama and I submitted a paper to *Nature* reporting the first patch clamp whole-cell recordings (WCRs) of current from an electrically nonexcitable cell type, the pancreatic acinar cell.^4^ We combined whole cell and single channel current recordings and thereby counted the number of high-conductance Ca^2+^-activated K^+^ channels in a single cell.^4^ In this study, we also used the Ca^2+^-activated channels as endogenous Ca^2+^ sensors to measure the cytosolic Ca^2+^ concentration.^4^ I was fully aware of work being carried out at that time, at the Max-Planck Institute for Biophysics in Frankfurt, showing that inositol 1,4,5-trisphosphate (IP_3_) released Ca^2+^ from intracellular stores in permeabilized pancreatic acinar cells, which was published in *Nature* late in 1983.^5^

The obvious experiment to do, at that point in time, would have been to use the WCR configuration to test directly the effect of intracellular IP_3_ application. This experiment was finally done in my laboratory, but only 5 years later.^6^ It showed that a constant level of IP_3_ evoked repetitive spikes of Ca^2+^-dependent current due to repetitive Ca^2+^ release from internal stores.^6^ If I had done this experiment in 1983, which would have been entirely feasible, the effect would have been very considerable. Ca^2+^ spiking because of IP_3_-elicited repetitive Ca^2+^ release in a mammalian cell would have been shown several years before Peter Cobbold and his collaborators demonstrated Ca^2+^ spiking evoked by hormonal stimulation in liver cells.^7^

Why did I delay doing this key experiment for such a long time? The reason is that I did not think it would work. These were still very early days in the history of the patch clamp technique and the first WCRs, which were carried out on the electrically excitable chromaffin cells by Alain Marty and Erwin Neher, indicated rapid equilibration of small ions between patch pipette and cell interiors.^8^ Given the enormous volume of the patch clamp pipette, as compared with the cell interior, I assumed (wrongly as it turned out) that in such experiments the Ca^2+^ concentration would be uniform throughout the cytosol and determined by the Ca^2+^ concentration in the pipette solution. Ca^2+^ buffers, usually EGTA, were of course always present in the patch pipette solutions and in general we, and others, assumed that the cytosolic Ca^2+^ concentration in WCR experiments could be calculated from the concentrations of Ca^2+^ and EGTA, taking into account the value of the Ca^2+^–EGTA dissociation constant at the known pH in the pipette solution.^8^

The (correct) image of the very large pipette and the very small cell was always with me and, like the doorkeeper in Kafka’s story, was so strong that it seemed hopeless even to contemplate doing an experiment that would depend on the ability of regions of the cell close to the Ca^2+^-sensitive channels to regulate Ca^2+^ independently of the bulk cytosolic Ca^2+^ concentration. A further analogy with Kafka’s parable is that, like the door to the Law that was specifically and exclusively provided for the man from the country, the IP_3_-WCR experiment was uniquely suited to the expertise and technology available at that time in my laboratory. In fact, and—to my luck—it turned out that even after a 5-year delay nobody else had attempted to do the experiment and so our paper, when it was finally published in 1989,^6^ still had impact. The results and conclusions were later confirmed by combined assessments of Ca^2+^-dependent currents and direct measurements of local changes in the cytosolic Ca^2+^ concentration in the subcellular region of secretory control.^9^^,^^10^

I grew up scientifically in a mostly very critical environment in the Institute of Medical Physiology at the University of Copenhagen, which in the late 1960s was dominated by several very clever individuals, who were very good at finding arguments against new ideas and experimental approaches. There is of course a place for such thinking, but one should never be intimidated. Even if one doubts that a key experiment is likely to work, there is merit in having the courage to try.
